# A Comparison of Iterative Reconstruction and Prone Imaging in Reducing the Inferior Wall Attenuation in Tc-99m Sestamibi Myocardial Perfusion SPECT

**DOI:** 10.4274/mirt.83007

**Published:** 2017-10-02

**Authors:** Duygu Kuşlu, Emel Öztürk

**Affiliations:** 1 Antalya Training and Research Hospital, Clinic of Nuclear Medicine, Antalya, Turkey; 2 Memorial Hospital, Clinic of Nuclear Medicine, Ankara, Turkey

**Keywords:** Myocardial perfusion SPECT, attenuation correction, prone positioning, iterative reconstruction

## Abstract

**Objective:**

Prone positioning, iterative reconstruction (IR-OSEM) and electrocardiography (ECG) gating have been demonstrated to improve the specificity of myocardial perfusion SPECT (MPS) in the diagnosis of coronary artery disease.

**Methods:**

The gated supine and prone MPS images of 45 patients were reconstructed with both IR-OSEM [supine (SIR) and prone (PIR)] FBPs [supine (SFBP), prone (PFBP)] for comparison. Perfusion, wall motion (WM) and wall thickening were also interpreted semi-quantitatively. Two groups were generated as those with normal or abnormal findings. Segmental myocardial tracer uptake values were noted from four of the reconstructed images from 17 segment model of bullseye.

**Results:**

The difference between mean values and the standard deviations of the % tracer uptakes of inferior wall segments were statistically significant in all images. The normalcy rates were highest in PIR images, followed by PFBP and SIR images. The number of patients with any perfusion abnormality were 42, 12, 32, and 6, in SFBP, PFBP, SIR and PIR images, respectively. The six patients with perfusion abnormality in PIR images were re-evaluated with rest images and were diagnosed with a fixed perfusion defect. There was positive correlation between WM and either PFBP or PIR images. Sixteen patients’ WM were evaluated as abnormal while only 6 patients’ perfusions were abnormal in PIR.

**Conclusion:**

Prone imaging in addition to a supine perfusion SPECT improves imaging quality of the inferior wall, especially when reconstructed with iterative methods. If prone imaging can not be performed, ECG-gating can also be used as a beneficial method.

## Abstract (tr)

**Amaç:**

Koroner arter hastalığı tanısında miyokard perfüzyon SPECT (MPS) görüntüleme spesifitesini artırmak için pron pozisyonlama, iteratif rekonstrüksiyon (IR-OSEM) ve elektrokardiyogram (EKG) tetikleme gibi yöntemler geliştirilmiştir.

**Yöntem:**

Bu yöntemleri birbirleriyle karşılaştırmak amacıyla, kombine gated supin ve pron Tc-99m MIBI MPS görüntülemesi yapılan 45 hastanın görüntüleri retrospektif olarak yeniden değerlendirildi. Her iki görüntüleme de IR-OSEM ile rekonstrükte edilerek [supin (SIR), prone (PIR)], rutin filtered-back projeksiyon (FBP) ile rekonstrükte edilmiş görüntülerle [supin (SFBP), prone (PFBP)] karşılaştırıldı. Perfüzyon, duvar hareketi (WM) ve kalınlaşması görsel olarak değerlendirilerek normal veya anormal olarak gruplandırıldı. Miyokardiyal segmental aktivite tutulum oranları 17 segment modeline göre değerlendirildi.

**Bulgular:**

İnferior duvar % aktivite tutulum oranlarında dört yöntemde anlamlı farklılık bulundu. Normallik oranı en yüksek PIR görüntülerde ve takiben sırasıyla PFBP ile SIR görüntülerdeydi. Perfüzyonu anormal hasta sayısı SFBP, PFBP, SIR ve PIR görüntülerde sırasıyla 42, 12, 32 ve 6 olarak değerlendirildi. Bu altı hasta istirahat görüntüleriyle birlikte tekrar değerlendirildiğinde sabit perfüzyon defekti tespit edildi. WM ile PFBP perfüzyon ve PIR perfüzyonu arasında korelasyon tespit edildi. WM anormal olan hasta sayısı 16 iken, PIR görüntülerde perfüzyonu anormal olan hasta sayısı 6 idi.

**Sonuç:**

Egzersiz sonrası MPS’ye eklenen pron görüntüleme, özellikle IR-OSEM ile rekonstrükte edildiğinde, inferior duvarda atenüasyonu belirgin azaltmaktadır. Pron görüntüleme uygulanamadığında EKG-tetikleme de yardımcı bir yöntemdir.

## INTRODUCTION

Although myocardial perfusion SPECT (MPS) imaging is accepted as the method of choice to evaluate coronary artery disease (CAD), tissue attenuation and reconstruction artifacts reduce the test’s specificity. Tissue attenuation artifacts are mostly located in the inferior wall in men and the anterior wall in women. Intense sub-diaphragmatic activity in the neighborhood of the heart also may produce artifacts (1,2). It can either mask a true perfusion defect (1,3), or cause false perfusion defects (3,4) due to fil-tered back projection (FBP) reconstruction that suppresses inferior wall counts adjacent to intense sub-diaphragmatic activity (5).

Various methods have been used to reduce artifacts in MPS, such as gated imaging and prone imaging. Prone imaging has been demonstrated to decrease inferior wall attenuation as well as improving the specificity rate (6,7). However, since prone imaging can cause false positive anteroseptal wall defects it needs to be combined with supine acquisitions (8,9,10). Our primary aim in this study was to compare the effectiveness of the methods performed to increase the specificity of the exam [gated- prone scans and iterative reconstruction (IR)] especially on artifact correction.

## MATERIALS AND METHODS

### Study Population

The supine and prone MPS images of 45 patients [42 male, 3 female, mean age: 58.76±11.84 years, mean body mass index (BMI): 28.5±4.2 kg/m^2^] were re-examined. Images with suboptimal image and gating quality were excluded.

### Stress Testing and Radiopharmaceutical Administrations

Patients underwent a 2-day protocol using Tc-99m MIBI for each study. When achievable, a symptom limited treadmill exercise test with Bruce protocol was applied, acquisition was started approximately 30 minutes after radiopharmaceutical administration. Adenosine stress was performed in 3 patients and infused at 40 mcg/kg/min for 4 minutes. Acquisition was started approximately 40 minutes after radiopharmaceutical administration.

All patients drank 200 mL milk or ate 100 mg chocolate 10 minutes after radiopharmaceutical injection to accelerate hepatobiliary excretion. All patients also drank 200 mL soda prior to imaging in order to decrease infra-diaphragmatic activity.

### Data Acquisition and Processing

Gated SPECT was performed with a dual-head camera (Millenium MG, GE Medical Systems, Milwaukee, WI, USA) equipped with high resolution collimators, with the following parameters for supine acquisition: 180 rotation arc, 64 projections, 25 s/projection, 8 frames/heart cycle, no arrhythmia rejection and 64x64 matrix. Prone imaging was performed immediately after supine image acquisition with 15 s/projection and with the same parameters without gating. Attenuation or scatter correction was not applied. Supine and prone images were reconstructed with both FBP (butterworth, frequency: 0.52, power: 5) [supine (SFBP), prone (PFBP)] and IR-OSEM [supine (SIR), prone (PIR)] by Cedars-Sinai QGS/QPS (Los Angeles, California) software in multiple color scales. MPS scans were viewed on a dedicated workstation (Xeleris; GE Healthcare) by using default reconstruction parameters in the standard format for display of tomographic cardiac studies.

### Image Interpretation

Semi-quantitative visual interpretation was performed from short-axis and vertical long-axis slices using a 17-segment model (11). Only the segments representing the inferior wall (segment number 4, 10, 15) and the apex (segment number 17) were scored by an experienced observer using a 5-point scoring system (0-normal, 1-equivocal, 2-moderate, 3-severe reduction of isotope uptake, and 4-absence of detectable tracer uptake). Scores 0 and 1 were accepted as normal. Since we did not aim to make a clinical diagnosis, the normality scores were accepted with this narrow range to be able to recognize any possible changes.

Quantitative myocardial isotope uptake percentages of all myocardial segments were obtained from the 17 segment bullseye model.

Visual wall motion (WM) [6-point scoring system (0-normal, 1-mild hypokinesia, 2-moderate hypokinesia, 3-severe hypokinesia, 4-akinesia, 5-dyskinesia)], and wall thickening (WT) [4-point scoring system (0-normal, 1-mild, 2-moderate to severe, 3-absent)] scoring of the inferior wall and apical segments was made only in supine images that allowed electrocardiography (ECG) gating (11).

### Statistical Analysis

All continuous variables were expressed as mean±SD. Repeated measures variant analysis was used to compare semi-quantitative interpretations (p<0.05), McNemar analysis was used to compare normalcy rates (p<0.05). Friedmann tests and correlation analysis was used to compare differences (SPSS Software, Version 20.0; SPSS Inc., Chicago, Illinois).

## RESULTS

### Patient Demographic Characteristics

There was a heterogeneous patient population regarding the likelihood of CAD; however mostly low likelihood of disease.

The differences of % tracer uptakes of all myocardial segments: In the inferior wall segments the difference (between % tracer uptakes) were significant in terms of all variables (p<0.01). In the apical segment (Seg. No. 17), the difference between SFBP, SIR and PIR in terms of % tracer uptakes were also significant (p=0.000), but not significant in PFBP and SIR images (p=0.314) (Table 1). In the anterior and septal wall segments the difference between PFBP and SIR in terms of % tracer uptakes was not significant similar to the apex, except segment 14 that indicates the septum in the short axis image of the apical section. Hereby PFBP and PIR counts were similar (p=0.405). Within the lateral wall segments, there were no differences between SFBP and SIR, PFB and PIR in terms of % tracer uptakes, except segment 16 that indicates the lateral wall in the short axis image of the apical section and in segment 17 that indicates the apex in only SFBP and SIR (p=0.404).

**Figure 1:**
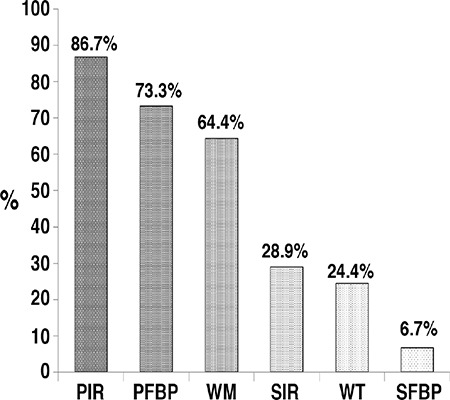
Normalcy rates of the groups

The results of the semi-quantitative perfusion interpretations of the inferior wall and apical segments: There were 42, 32, 12 and 6 patients who were evaluated as abnormal in SFBP, SIR, PFBP, and PIR images, respectively. Thus, the highest normalcy rate was in PIR images with 86.7%, followed by PFBP images with 73.3%, and SIR images with 28.9% (Figure 1, Table 2). The differences between the groups in terms of normalcy rates were significant (p<0.05). The rest images of the 6 patients who have been evaluated as abnormal in stress PIR images were re-evaluated and the summed difference scores were calculated to be ‘0’, which indicates a fixed perfusion defect.

There were 29 (64.4%) and 11 patients (24.4%) that normal regional WM and WT, respectively, as the WM and WT interpretations were made semi-quantitatively only in inferior and apical segments (Figure 1). In daily practice, we evaluate myocardial functions often while interpreting a patient’s images. To compare the differences and consider the similarities of the methods, we performed a correlation analysis between functional data, prone FBP and IR-OSEM. There was a significant but weak correlation between WM and PFBP (p=0.008, r=0.392), and a significant and moderate correlation between WM and PIR images (p=0.000, r=0.528) (Table 3). Although there were 16 patients with abnormal WM, perfusion was considered as abnormal in only 6 patients in PIR. There was also a significant but weak correlation between WT and SIR images (p=0.031, r=0.322) (Table 3). When we compared WM and WT scores with the related perfusion scores in the segment base; the most powerful correlations were detected between WT and PFBP Seg. No. 17 (p=0.000, r=0.863), SIR Seg. No. 15 (p=0.000, r=0.744), SFBP Seg. No. 15 (p=0.000, r=0.706), as well as between WM and SFBP Seg. No. 17 (p=0.000, r=0.626), and PIR Seg. No.15 (p=0.000, r=0.588) (Table 4).

## DISCUSSION

Our study is thought to be unique in comparing different methods being used to detect attenuation artifacts of the inferior wall (supine vs. prone positioning, IR-OSEM vs FBP and ECG gating methods) in MPS. Images included in this study were the patients’ with supine gated MPS which have inferior wall perfusion abnormality, requiring prone imaging. The primary aim of this study was to compare the supine images with prone images in two different projection methods (FBP and IR-OSEM) with a secondary aim to correlate this data with functional scores.

In this study, the percentage of inferior wall segments tracer activities were significantly high in PIR, followed by PFBP (p<0.01). Normalcy rates were also the highest in PIR (86.7%) followed by PFBP images (73.3%). Among regional WM and thickening assessments of the inferior wall and apex, the performance of the WM scores was better than the thickening scores (normalcy rates 64.4% vs 24.4%, respectively). The correlation was significant but poor between WM and PFBP (p=0.008, r=0.392), while there was a significant and moderate correlation between WM and PIR images (p=0.000, r=0.528). The results indicate that prone imaging and IR-OSEM reconstruction have similar impacts on the % tracer uptake by the anterior and septal walls, prone imaging with statistically insignificant higher count rates. However, prone imaging significantly increases the counts of the lateral wall. Count rates of the segments were higher in IR-OSEM as compared to FBP, and in prone images as compared to supine images. Nevertheless, the highest counts were obtained from PIR images in all segments except in segment No. 16.

One of the most common problems with perfusion SPECT is the decrease in the radiotracer uptake due to several artifacts, thus reducing its specificity rate in detecting CAD. Various methods have been suggested to overcome this obstacle.

Katayama et al. (12) reported the sensitivity rate of the supine stress-rest Tl-201 images to be higher than stress-combined supine-prone images (77% vs 55%). However, prone imaging has been shown to improve the accuracy of diagnosing CAD of the inferior wall (71% vs 83%). Some authors have suggested that prone imaging improved the accuracy for diagnosing CAD without decreasing the sensitivity rate (7,10,12).

Reconstruction with IR-OSEM enabled reduction in attenuation correction (AC), thus provided modest improvement in the diagnostic accuracy. When AC (IR + AC) was added, a good correlation with PET in diagnostic accuracy was obtained as well as in stress perfusion scores (13). Qutub et al. (13) evaluated a new RR reconstruction algorithm for equivocal SPECT MPS. IR showed better results than FBP with the frequency of ‘‘perceived’’ non-equivocal studies. Bai et al. (14) have shown that OSEM significantly decreased the count-loss artifact in both the inferior and posterior walls.

Evaluation of left ventricular functional parameters from gated scans with cinematic display mode increases the diagnostic accuracy, hereby, true perfusion defects can be separated from the artifactual defects (15). Our findings were in accordance with these results, the normalcy rate increased to 64.4% with WM analysis in this study. However, it should be taken into account that in case of sub-endocardial infarction, a true myocardial perfusion defect can be false-negatively interpreted as soft-tissue attenuation since there may not be any functional abnormalities (16).

Another technique that has been shown to reduce this problem is nonuniform AC, unfortunately requires specialized hardware and software and, in some implementations, may overcorrect the inferior wall, leading to lower sensitivity for detection of a true perfusion defect (17,18).

In their study on comparing computed tomography-based AC vs. prone scanning, Malkerneker et al. (19) reported that prone imaging added to a supine imaging with and without AC did not significantly reduce the equivocal evaluation numbers. Prone imaging and AC were more helpful in men as compared to women. However, they used FBP for supine and OSEM for AC images.

Although prone imaging requires additional acquisition time, it is easy to perform since it does not require any dedicated system/software. The prone posture itself displaces the diaphragm downward, thus reducing diaphragmatic attenuation. In addition, it enables the heart to come closer to the imaging table, indirectly limiting patient movement during acquisition (6,7,20,21). Patients with inferior wall perfusion abnormality on normal prone images have been shown to have an excellent and comparable prognosis to patients with normal supine alone myocardial perfusion (10). Supine imaging is advised as the ‘‘standard’’ imaging protocol for MPS by ASNC. In case of suspicion, combined protocols followed by post-stress prone imaging can be a useful ‘‘option’’ to differentiate perfusion abnormalities caused by artifacts (22).

Several studies reported that prone imaging increased false positive anteroseptal and lateral wall defects (7,23). Slomka et al. (9) reported no difference in normalcy rates between prone and supine acquisitions in women. Our study findings also did not support aforementioned studies. In our study, the count rates were higher in IR-OSEM than FBP, and in prone images than supine images. However, the highest counts were obtained from PIR images in all segments except segment No. 16. To the best of our knowledge, there are no studies evaluating the effect of IR-OSEM methods on myocardial perfusion. This study needs to be improved by including coronary angiography (CAG) results to evaluate the sensitivity, specificity, negative and positive predictive values of these methods.

### Study Limitations

We did not compare the diagnostic accuracy of SPECT MPS with CAG. This is a small, single center study. Though few patients had subsequent invasive CAG and therefore anatomical data does not exist to compare.

Due to the low number of female patients, gender difference could not be evaluated.

We didn’t compare the effect of mean BMI, since we think obese patients were more likely to be interpreted as equivocal. Nevertheless, it has been shown that BMI does not significantly effect equivocal interpretation numbers (19).

Semi-quantitatively, we focused on inferior defects. However, it has been shown that most perfusion defects are located in the inferior wall in men, and to a lesser extent in the anterior wall in women (19).

Another limitation of this study is the heterogeneous patient population including those with a relatively low likelihood of CAD, those with known CAD, and those who underwent coronary bypass grafting and percutaneous coronary interventions.

## CONCLUSION

Prone imaging added to a stress MPS significantly improves the inferior wall attenuation artifact, especially when reconstructed with IR-OSEM. Since prone imaging and WM is well correlated, WM analysis can be used as a helpful method if prone imaging cannot be performed.

## Figures and Tables

**Table 1:**
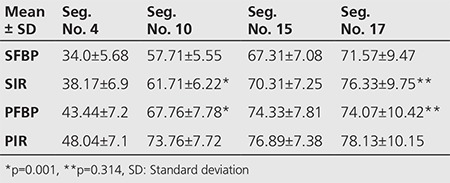
Mean ± standard deviations and comparisons of the % tracer activities of the inferior wall and apical segments

**Table 2:**
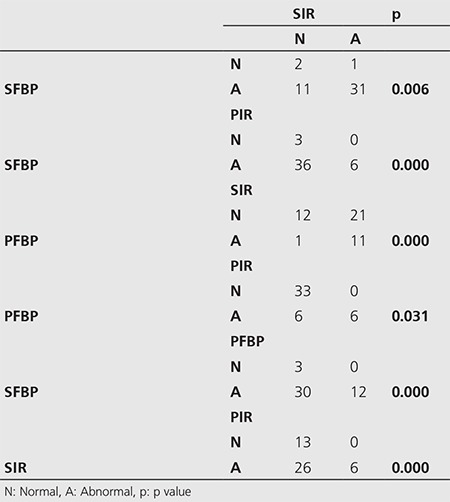
Comparison of the normalcy rates between groups (McNemar, p<0.05)

**Table 3:**
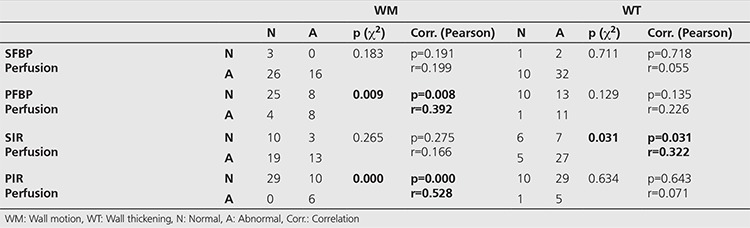
Distribution of the number of patients according to wall motion and wall thickening scores considered as normal/abnormal and their statistical comparisons (χ^2^, p<0.05)

**Table 4:**
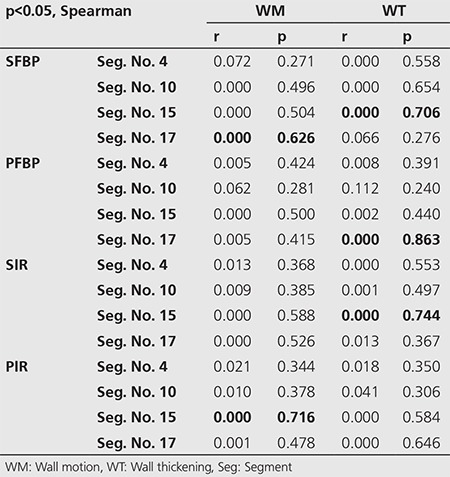
Correlation analysis of wall motion and wall thickening scores according to segments (p<0.05 Spearman)
